# Orbital Desmoid-Type Fibromatosis: A Case Report and Literature Review

**DOI:** 10.1155/2018/1684763

**Published:** 2018-03-08

**Authors:** Alessandro Moro, Paolo De Angelis, Giulio Gasparini, Sandro Pelo, Gianluigi Petrone, Emanuela Lucci Cordisco, Umberto Garagiola, Giuseppe D'Amato, Gianmarco Saponaro

**Affiliations:** ^1^Department of Oral and Maxillofacial Surgery, Catholic University of the Sacred Heart Medical School, Rome, Italy; ^2^Department of Histopathology and Cytopathology, Catholic University of the Sacred Heart Medical School, Rome, Italy; ^3^Department of Genomic Medicine, Catholic University of the Sacred Heart Medical School, Rome, Italy; ^4^Department of Orthodontics, University of Milan, Milan, Italy

## Abstract

**Purpose:**

Desmoid-type fibromatosis is a benign fibrous neoplasia originating from connective tissue, fascial planes, and musculoaponeurotic structures of the muscles. Currently, there is no evidence-based treatment approach available for desmoid fibromatosis. In this article, a case of a patient in the pediatric age affected by desmoid fibromatosis localized in the orbit is presented. The aim of the article is to describe this unusual and rare location for the desmoid fibromatosis and outline the principle phases in the decision-making process and the therapeutic alternatives for a patient affected by desmoid fibromatosis.

**Methods:**

The protocol of this review included study objectives, search strategy, and selection criteria. The primary end point of this study was to analyze the head and neck desmoid fibromatosis. The secondary end point was to identify the available therapies and assess their specific indications.

**Results:**

The mean age of patients was 18.9 years ranging from 0 to 66, and 52% were female. A bimodal age distribution was observed, and two age peaks were identified: 0–14 years (57%) and 28–42 years (18%). The most common involved areas were the mandible (25%) followed by the neck (21%). In 86% of the cases, the treatment was the surgical resection of the disease, and only in 5% of the cases, the surgical resection was followed by adjuvant radiotherapy.

**Conclusion:**

The orbital location is extremely rare, especially in the pediatric population. The management of desmoid fibromatosis is based on the function preservation and the maintenance of a good quality of life, but in case of symptomatic patients or aggressive course of the disease or risk of functional damages, the surgical approach may be considered. Therapeutic alternatives to surgical resection are radiotherapy and systemic therapy.

## 1. Introduction

Desmoid-type fibromatosis is a benign fibrous neoplasia originating from connective tissue, fascial planes, and musculoaponeurotic structures of the muscles [1]. This tumor is not capsulated and usually infiltrates along fascial planes and invades adjacent neurovascular structures [2]. In 1832, McFarlane was the first to describe a case, and six years later, Muller coined the term “desmoid tumor,” derived from desmos which means tendon-like in Greek [3, 4]. This tumor is extremely rare, and the incidence of desmoid fibromatosis is 2 to 4 per 1 million per year with a female-to-male ratio of 3 to 1 [5–8]; it represents less than 3% of all soft tissue sarcomas [9]. The disease can be divided into two groups because desmoid fibromatosis can be sporadic or associated with a hereditary syndrome. In both groups, there is a genetic predisposition [8]. The incidence of desmoid fibromatosis is remarkably higher in patients affected by familial adenomatous polyposis and Gardner syndrome [10]. In these cases, the disease is usually intra-abdominal. Another described hereditary syndrome involved is the autosomaldominant inheritance of familial infiltrative fibromatosis [11]. In the familial adenomatous polyposis, Gardner syndrome, and familial infiltrative fibromatosis, lesions are associated with the inactivation of the APC tumor suppressor [6, 12]. In the sporadic desmoid fibromatosis, more than 60% of tumors contain mutations in *CTNNB1* (the gene that codes for beta-catenin) [12] with p.T41A (threonine to alanine), p.S45F (serine to phenylalanine), and p.S45P (serine to proline) being the most frequent [13, 14]. Desmoid fibromatosis is derived from mesenchymal stem cells in the deep soft tissues, and mutations of beta-catenin can support tumorigenesis causing resistence to the inhibitory influence of APC and maintaining mesenchymal progenitor cells in a less differentiated state [6, 12]. Accumulation of beta-catenin can be detected using immunochemistry, but limitations have been acknowleged in this diagnostic procedure [13]. Abdominal desmoid fibromatosis is slightly more frequent than extra-abdominal desmoid fibromatosis [7]. Predilected sites of the extra-abdominal desmoid fibromatosis are the shoulder, chest wall and back, thigh, and head and neck [6, 15]. Head and neck lesions represent about 12% to 15% of desmoid fibromatosis [16]. In the paediatric population, these lesions have an equal sex incidence, most are extra-abdominal and may be multifocal [6, 17]. The incidence of childhood desmoid fibromatosis presents two age distribution peaks: one early at approximately 4.5 years and a second between 15 and 35 years [7, 16]. The pathogenesis of desmoid fibromatosis is multifactorial and includes genetic predisposition, endocrine factors, and physical factors such as trauma [6]. These tumors are characterized by infiltrative growth and a tendency toward local recurrence but an inability to metastasize [6, 18]. Clinically, the extra-abdominal fibromatosis appears as a deep-seated and circumscribed mass growing insidiously, often with no pain [6] and a variable clinical course [9]. Currently, there is no evidence-based treatment approach available for desmoid fibromatosis [9]. In the past, the first-line treatment was the surgical resection with negative margins, but there is a reported recurrence rate of 15% to 77% [16, 19], and also in the case of adjuvant radiation therapy, the local control is reached in about 75% of the cases [2, 17, 19]. Recently, the high risk of relapse and the necessity to reach function preservation and a good quality of life have caused the diffusion of a watch-and-wait approach in the first phase. Radiotherapy alone may serve as a primary therapy and cause minor deficits in those patients affected by unresectable tumors, or it may serve as adjuvant therapy. Other nonsurgical approaches have been introduced as a primary approach in case of inoperable patients or as adjuvant therapy, both with variable results [17, 19, 20].

In this article, a case of a patient in the pediatric age affected by desmoid fibromatosis localized in the orbit is presented. The aim of the article is to describe this unusual and rare location for the desmoid fibromatosis and outline the principle phases in the decision-making process and the therapeutic alternatives for a patient affected by desmoid fibromatosis.

## 2. Methods

The protocol of this review included study objectives, search strategy, and selection criteria. The primary end point of this study was to analyze the head and neck desmoid fibromatosis. The secondary end point was to identify the available therapies and assess their specific indications. Retrieval of studies was performed through MEDLINE on PubMed using keywords “extra-abdominal desmoid fibromatosis” and “head and neck desmoid fibromatosis” as free text word and/or combined with “therapy” and “treatment.” Initial searches were performed in March 2016, and only studies published as full papers in English from 1978 to 2016 in peer review journals were selected. Selection criteria were study design and the presence of sufficient data about sex, age, disease location, and treatment.

## 3. Results

After initial screening, 128 studies were identified, but the subsequent evaluation for eligibility has caused the exclusion of 22 studies because they were published only in abstract form, they were not in English, or they were not providing information about one or more of the requested areas (sex, age, disease location, and treatment). In total, 106 studies were considered adequate for this review.

The mean age of patients was 18.9 years ranging from 0 to 66, and 52% were female. A bimodal age distribution was observed, and two age peaks were identified: 0–14 years (57%) and 28–42 years (18%). The most common involved areas were the mandible (25%) followed by the neck (21%). In 86% of the cases, the treatment was the surgical resection of the disease, and only in 5% of the cases, the surgical resection was followed by adjuvant radiotherapy.

## 4. Case Report

**Figure 1:**
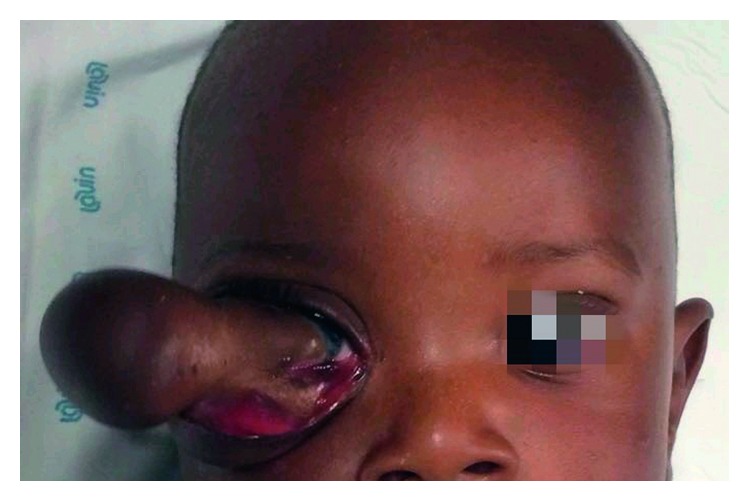
A painful and firm mass at the level of the right orbital area of substantial size in a male Senegalese ten-month-old baby.

**Figure 2:**
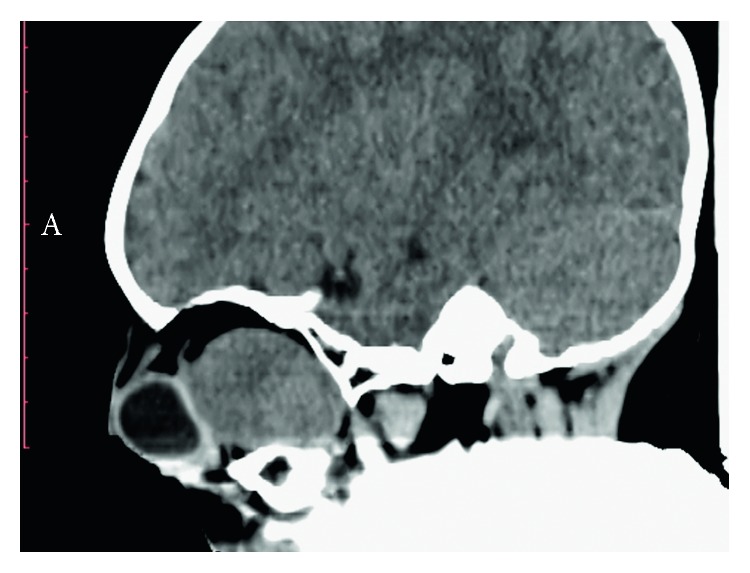
CT scan showing the orbital involvement with the lateral wall of the orbit infiltration.

**Figure 3:**
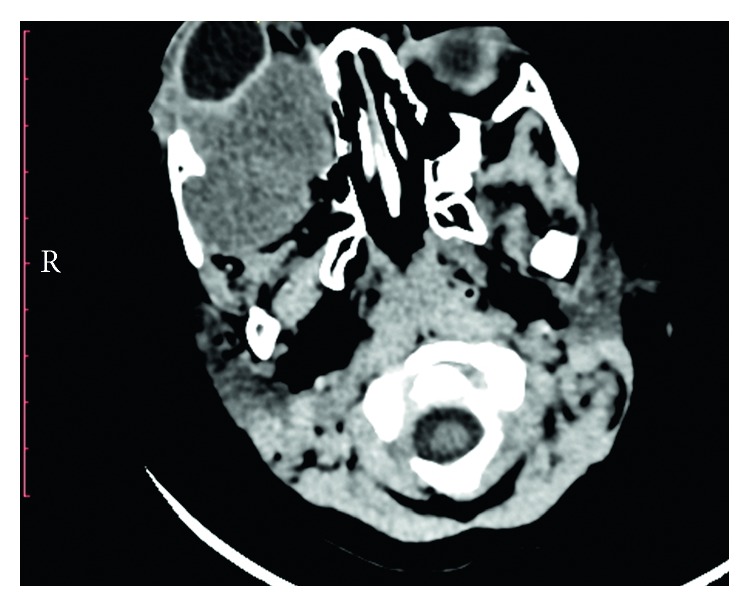
CT scan showing the neoplasm extended into the maxillary sinus and the temporal and infratemporal fossa.

A male Senegalese ten-month-old baby was referred to our department due to the presence of a painful and firm mass at the level of the right orbital area of substantial size. This neoplasm was first noticed three months after the birth as a swelling with exophthalmos and had also resulted in the progressive loss of visual acuity. In the following months, the neoplasm had met with a rapid expansive and infiltrative growth (Figure 1). Radiographic evaluation through a CT scan was performed. The computed tomography showed the orbital involvement with the lateral wall of the orbit infiltration. Furthermore, the neoplasm extended into the maxillary sinus and the temporal and infratemporal fossa (Figures 2 and 3). The patient had undergone several biopsies prior to the surgical intervention, and none of them was suggestive of desmoplastic fibroma but of other benign lesions (e.g., lipoma).

**Figure 4:**
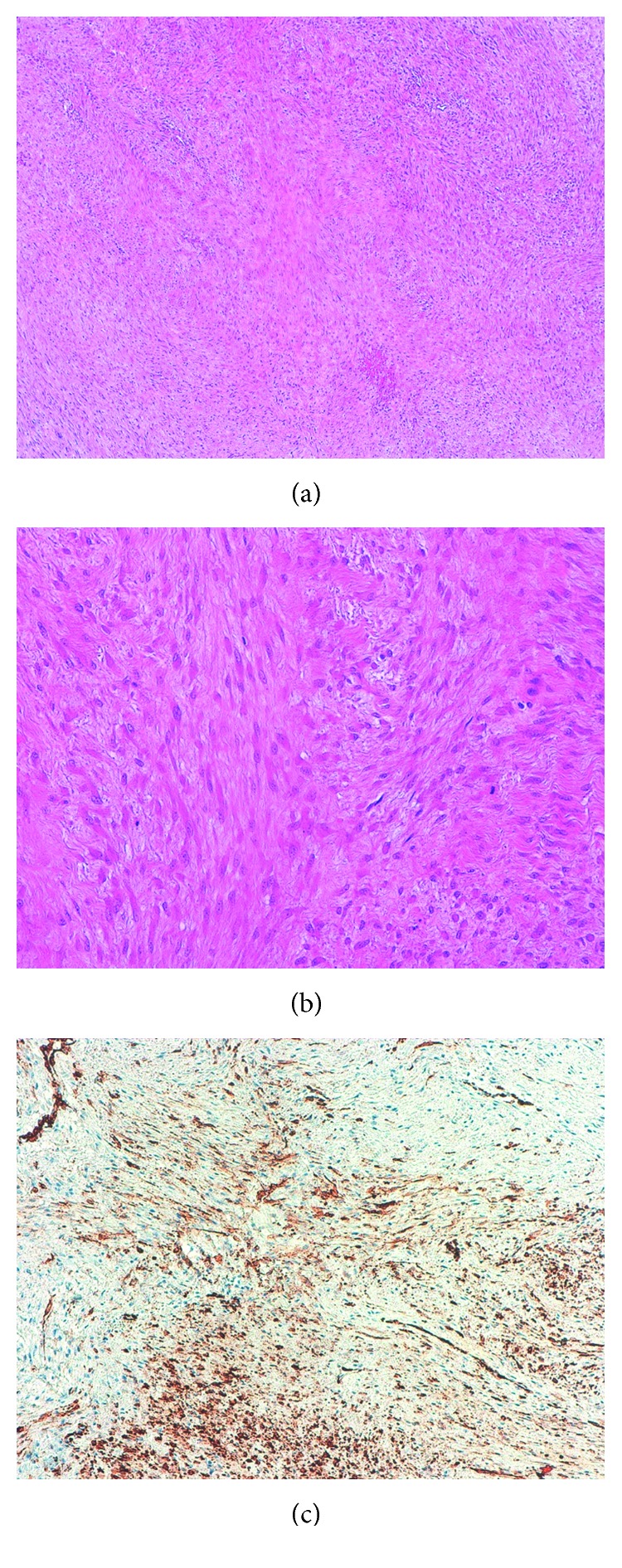
Histological features of desmoid fibromatosis.

**Figure 5:**
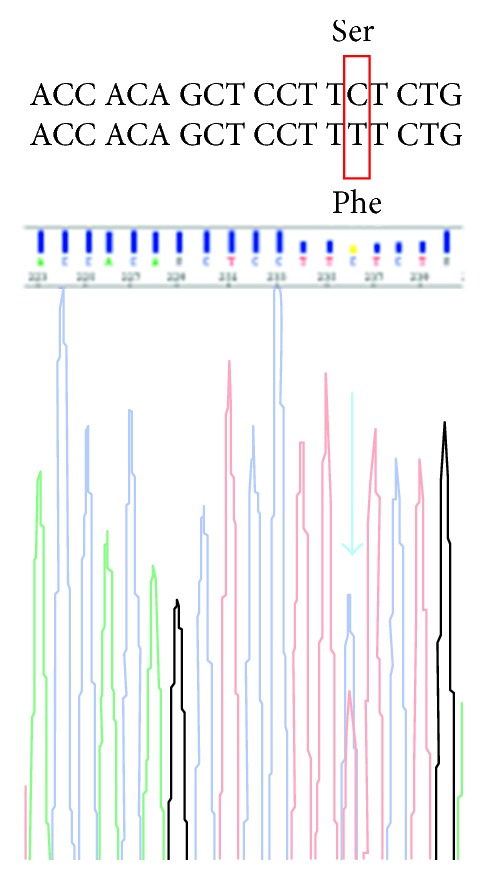
Mutational status of the catenin *β*-1 (*CTNNB1*) gene in the tumor analyzed by Sanger sequencing.

As it came to our attention, a biopsy of the neoplasm under general anesthesia was scheduled. The surgery was immediately carried out by our department. A transconjunctival incision was performed, and after creating an access to the neoplasm, a biopsy with the removal of the portion projecting to the outside of the eyelid was performed. Histopathological evaluation revealed the presence of fusiform cellular elements arranged in bundles, with low cell density. Moreover, there were areas of fibrosis with deposition of coarse hyalinized collagen bundles, and the optic nerve was unharmed. Although the location was unusual and rare, the histopathological evaluation was confirming the initial clinical suspect of “soft tissue sarcoma” revealing the presence of a desmoid-type fibromatosis. The histopathological evaluation revealed paucicellular fibrous proliferation in interlacing fascicles of bland fibroblasts (Figure 4(a), haematoxylin and eosin, original magnification 40x). High-power view showed uniform spindle-shaped cells with wavy, vesicular nuclei with minute nucleoli and indistinct cytoplasm separated by abundant collagen, rarely hyalinazed. Few mitotic figures and no atypia were observed (Figure 4(b), haematoxylin and eosin, original magnification 200x). Immunohistochemically, the spindle cells focally stain with smooth muscle actin (Figure 4(c), haematoxylin counterstaining, original magnification 100x). Subsequently, the mutational status of the catenin *β*-1 (*CTNNB1*) gene in the tumor was analyzed by Sanger sequencing, which revealed the S45F somatic mutation (Figure 5).

**Figure 6:**
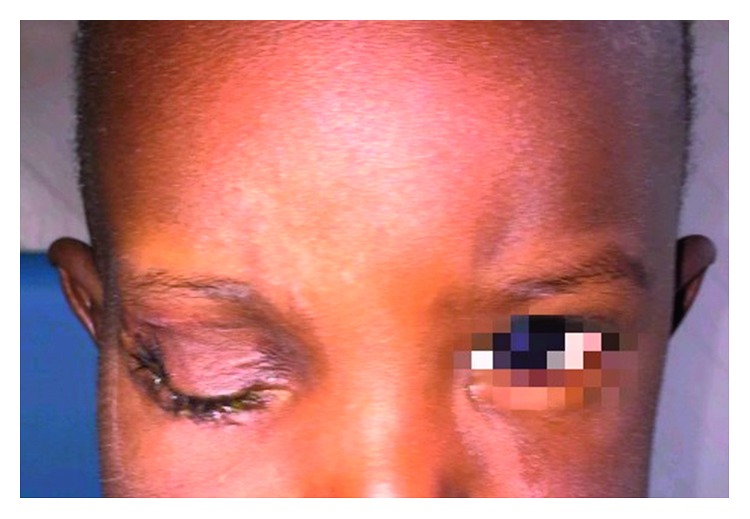
Postoperative period and tissue healing after the orbital exenteration with surgical resection of the tumor.

**Figure 7:**
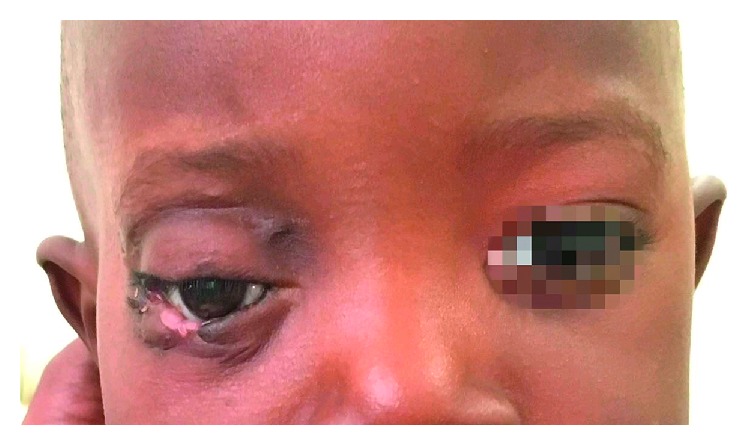
Application of an ocular prosthesis after the fornix deepening and the reconstruction of the lower eyelid.

Thus, the patient underwent orbital exenteration with surgical resection of the tumor. The surgical resection was performed by affecting the upper and lower eyelid fornix, and being the third side of the lower eyelid adherent to the neoplasm, it was removed. Later, the orbital content was resected together with the neoplasm. An excision with negative margins was achieved. The neoplasm, in addition to affecting the endorbital area, had infiltrated the lateral wall of the orbit and also extended into the maxillary sinus and the temporal and infratemporal fossa. The reconstruction of the orbital wall was obtained by means of a right temporal muscle flap using orbital access. The temporal muscle flap was rotated within the orbital cavity through the continuous solution of the orbital lateral wall. The postoperative period has elapsed without any complication (Figure 6). After the tissue healing was reached in order to correct the results of previous surgery of orbital exenteration, the fornix deepening and the reconstruction of the lower eyelid were performed using a myocutaneous laterally based upper eyelid flap with an inferolateral peduncle. The flap was partially deepithelized, rotated, and tunneled to reconstruct the lateral third of the lower eyelid. Furthermore, several mucosal grafts, taken from the right cheek region and the lower lip, were used as lining for the internal surface of the flap, and a retainer was applied. After a short retention period, the application of an ocular prosthesis has been scheduled (Figure 7). Follow-up of the patient has been performed for 40 months.

## 5. Discussion

Desmoid fibromatosis is a rare neoplasia, and the first challenge for the clinician in the treatment of this disease is the diagnosis. The correct diagnostic process is based on clinical, radiological, and histological evaluation [8]. For the pediatric population, the mandible is the most frequently affected site in the head and neck district [21, 22]. Orbital location is unusual and rare for the desmoid fibromatosis, and only four cases had been described in literature [23–26]. Differential diagnosis in the pediatric population and in this anatomical location can be done with cranial/nodular fasciitis, low-grade fibromyxosarcoma, low-grade myofibroblastic sarcoma, lipofibromatosis, malignant peripheral nerve sheath tumor, myofibroma, leiomyosarcoma, inflammatory myofibroblastic tumor, and hypertrophic scar [13, 27]. The main clinical characteristic of this tumor is the infiltrative growth that can involve muscles, soft tissues, and neurovascular structures. The radiological evaluation of the lesion can be done through computed tomography, but for the head and neck region, magnetic resonance imaging is preferred [8]. Desmoid fibromatosis is characterized by nonenhancing low signal intensity bands on magnetic resonance images. Biopsy is mandatory to confirm the clinical suspect of desmoid fibromatosis. For a correct diagnosis, it is important to remember that different histologic variations can be present, and seven morphologic patterns have been identified: conventional, hyalinized/hypocellular, staghorn vessel, myxoid, keloidal, nodular fasciitis-like, and hypercellular [8, 28]. Immunochemistry can be used to detect accumulation of beta-catenin and can be useful for the differential diagnosis [29]. In a study by Dômont et al. the 5-year recurrence rate reported was significantly higher for desmoid fibromatosis with beta-catenin mutation [30] and in particular in case of S45F mutation [31].

Nowadays, the treatment of desmoid fibromatosis is still debated because the evolution of the disease is unpredictable and stabilization or even regression is possible in 5–10% of the cases [9, 13]. In the management of this disease, a multidisciplinary approach is necessary to evaluate all the treatment alternatives available and choose between them [29]. The main problem is the high propensity to recur after initial surgical treatment, and in the head and neck district, the 5-year local recurrence rate reported by van Broekhoven et al. is 25% [32]. Influence of the surgical margin status on the recurrence rate is controversial for local control [8, 32–35]. In a report from Leithner et al. a 27% recurrence rate of extra-abdominal desmoid fibromatosis was reported in case of excision with negative margins, while it was 72% in case of intralesional excision [36]. In the recent past was recommended to attempt a function-sparing surgery with no macroscopic residual disease because trying to achieve negative microscopic margins in all patients could cause unnecessary morbidity [8]. Thus, the choice of a complete surgical resection was also influenced by the risk of causing considerable esthetic and functional deficits [19]. In the management of desmoid fibromatosis, function preservation and quality of life are considered fundamental goals to be reached because this is a nonmalignant neoplasia and there is not a risk of dedifferentiation in case of relapses [8, 9]. For patients with microscopic or macroscopic residual tumor, postoperative adjuvant therapy can be considered in order to obtain progression-free survival [19]. Furthermore, in some cases of desmoid fibromatosis, surgery can probably cause the release of growth factors during the wound healing that could act as a tumor enhancer increasing the risk of recurrence [8]. For these reasons, surgery as first-line treatment is under debate. Nowadays, the approach to aggressive fibromatosis is changing, and therapeutic alternatives to surgical resection are encouraged by the literature. A watch-and-wait policy is currently agreed as the initial strategy in case of asymptomatic lesions, absence of marked progression of the disease, and a non-life-threatening site because this approach could avoid unnecessary morbidity [37]. Furthermore, head and neck desmoid fibromatosis has a better prognosis [38]. The progression of the disease usually happens within the first 2 years of observation; thus, remaining stable for this period is a positive prognostic factor [8]. In the presented case, a watch-and-wait approach could not be adopted because the desmoid fibromatosis was symptomatic and was having an aggressive course, growing rapidly, and infiltrating the adjacent anatomical structures such as the lateral orbital wall, maxillary sinus, and temporal cavity and infratemporal fossa. Moreover, the neoplasia had already caused the loss of visual acuity in a short period of time. In this case, the rapid progression of the disease and the functional damages were discriminating factors in the choice of rejecting a watch-and-wait policy. The possibility to perform an eyelid-sparing orbital exenteration and the surgical resection of the neoplasm with immediate reconstruction using a temporal muscle flap was valued; a function-sparing intervention was planned after the histopathological analysis and the confirmation of an aggressive and rapid course of the disease. Orbital exenteration is a radical procedure consisting of removal of the orbital contents including orbital fat, conjunctival sac, globe, and part or all of the eyelids [39]. Eyelid-sparing techniques have been introduced as modification of exenteration with the aim of reaching better facial rehabilitation [39]. The main goals of reconstruction techniques are to restore the boundaries between the orbit and surrounding cavities and obtain aesthetic outcomes [40]. In the presented case for the reconstruction of the orbital wall was used a right temporal muscle flap using orbital access. This therapeutic option is characterized by its completion in one operative stage, short operative time, minimal donor site morbidity [41], and low risk of secondary infection.

In the presented case, it was possible to obtain a R0 resection. Adjuvant and postoperative radiotherapy was considered not indicated because of the age of the patient, the affected area, and the achievement of microscopic negative margin resection.

In pediatric patients, radiotherapy is associated with complications such as growth abnormalities and fractures, soft tissue injury (cellulitis and necrosis), long-term cosmetic and functional morbidity, and secondary malignancy [17]. Thus, the use of radiotherapy in the pediatric population is controversial and should be avoided because of both short- and long-term complications.

In adult patients, therapeutic alternatives to surgical resection in case of nonresectable lesions and in situations where surgical treatment would lead to functional impairment are radiotherapy and systemic therapy [9, 20], but due to the rarity of desmoid fibromatosis, there are no evidence-based data yet [9]. Radiotherapy as single-modality treatment seems effective for reaching local control, but Keus et al. report a complete regression of the disease only in 13.6% of the cases [20]. Tumor size can influence the local control of the disease [30]. Recommended dose for the radiotherapy is 50–56 Gray [8]. Doses over 56 Gray are not necessary and can cause an increased risk of radiation-related complications [42]. Radiotherapy as adjuvant treatment is able to significantly reduce the local recurrence rate [43].

Radiotherapy should be recommended for patients with nonradical tumor resection after primary surgery or at first recurrence and for function preservation. Furthermore, radiotherapy as adjuvant treatment improves local tumor control and should be preferred to radiotherapy at recurrence [43].

Currently, there are also different pharmacological options available: antihormonal therapies (tamoxifen), nonsteroidal anti-inflammatory drugs (NSAIDs), low-dose chemotherapy (methotrexate plus vinblastine), tyrosine kinase inhibitors (imatinib or sorafenib), and full-dose chemotherapy using anthracycline-based regimens, including liposomal doxorubicin [9]. In a pediatric population, the response rate after administration of a low dose of methotrexate and vinblastine reported by Skapek SX was 31%. This combination seems to be effective in promoting tumor regression or blocking tumor growth and is tolerated by children [44].

## 6. Conclusion

Desmoid-type fibromatosis is a benign fibrous neoplasia which can involve the head and neck district in the pediatric population causing considerable esthetic and functional deficits and affecting the patients' quality of life. The orbital location is extremely rare, especially in the pediatric population, and due to the infiltrative growth, it can involve the adjacent anatomical structures and cause the loss of visual acuity. The management of desmoid fibromatosis is based on the function preservation and the maintenance of a good quality of life, and for these reasons, the watch-and-wait policy is currently agreed as the initial strategy. In the pediatric population, the use of different pharmacological options should be considered in case of symptomatic patients or aggressive course of the disease or risk of functional damages. The surgical approach should be avoided if possible. A therapeutic alternative to systemic therapy and surgical resection is radiotherapy. However, radiotherapy is considered not indicated in the pediatric population and in the orbital location.
